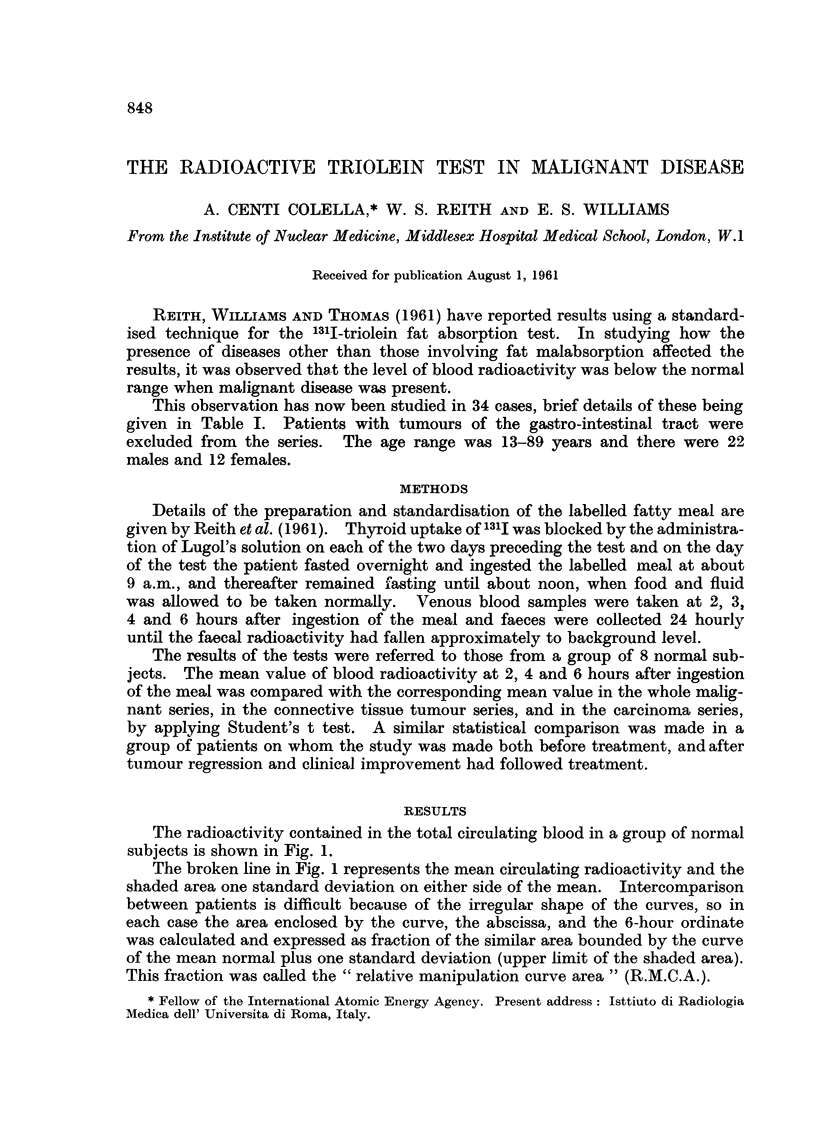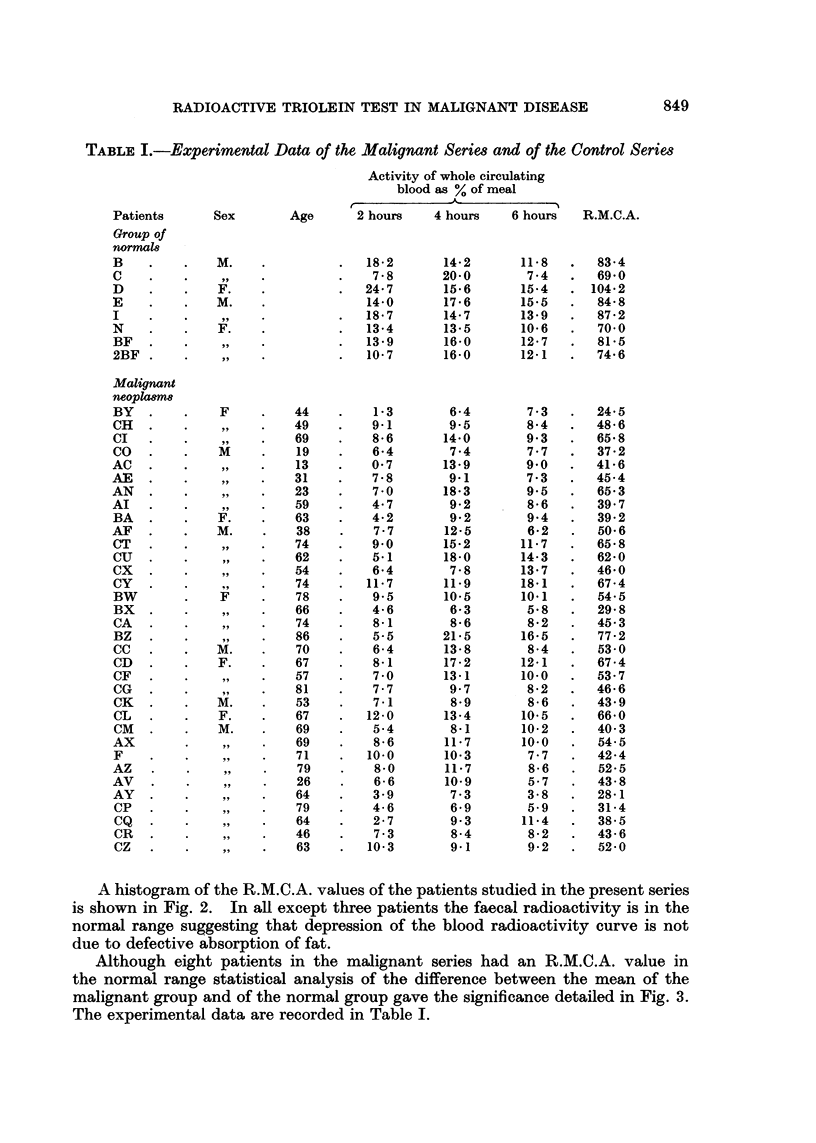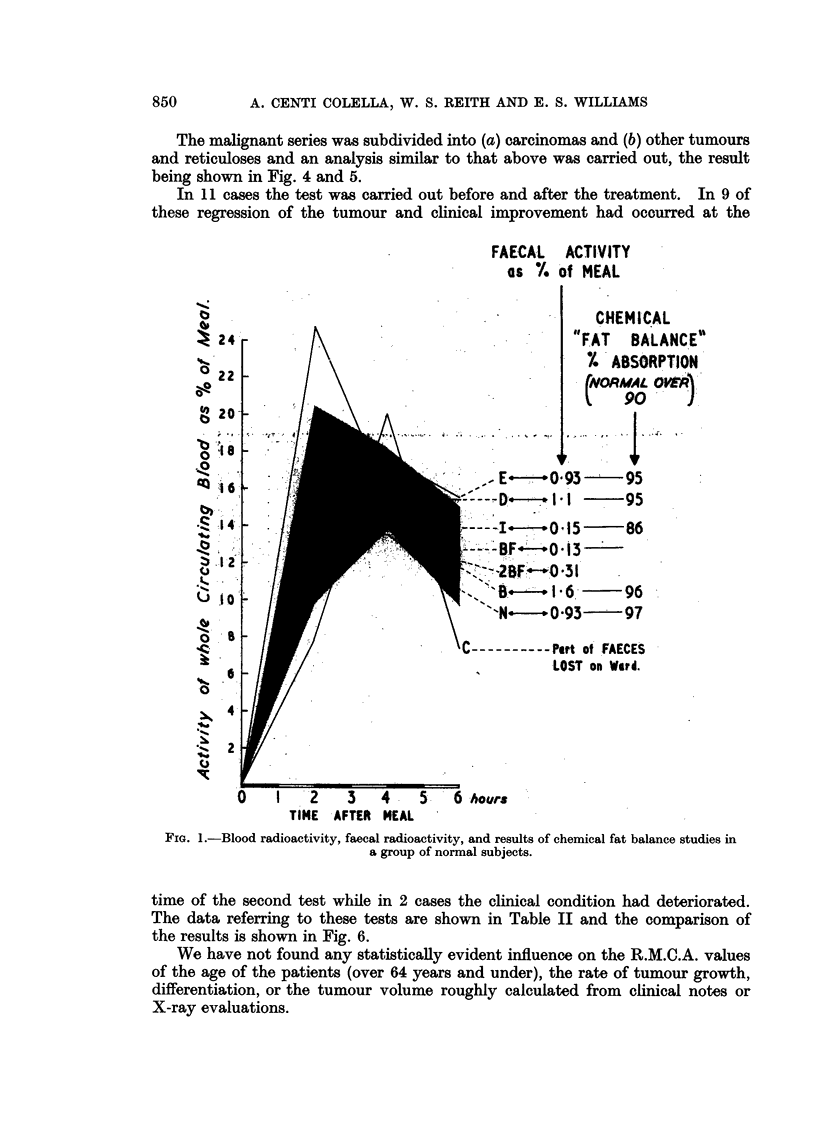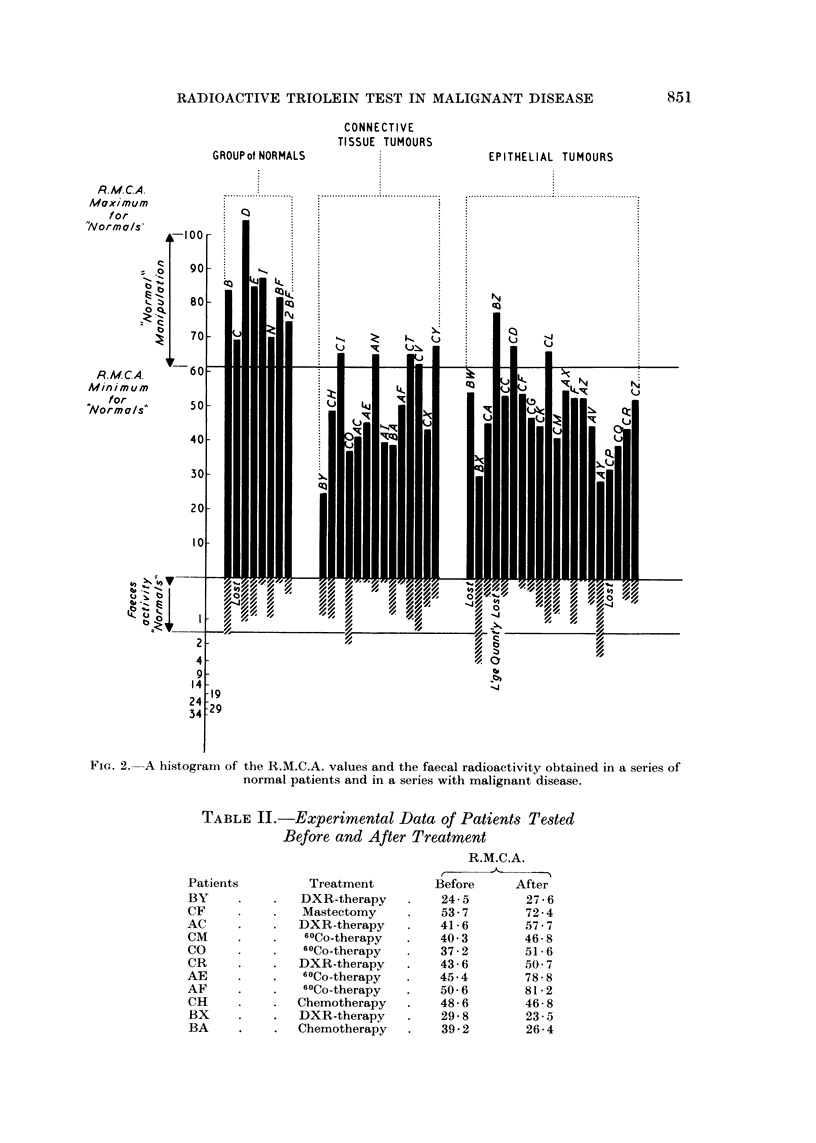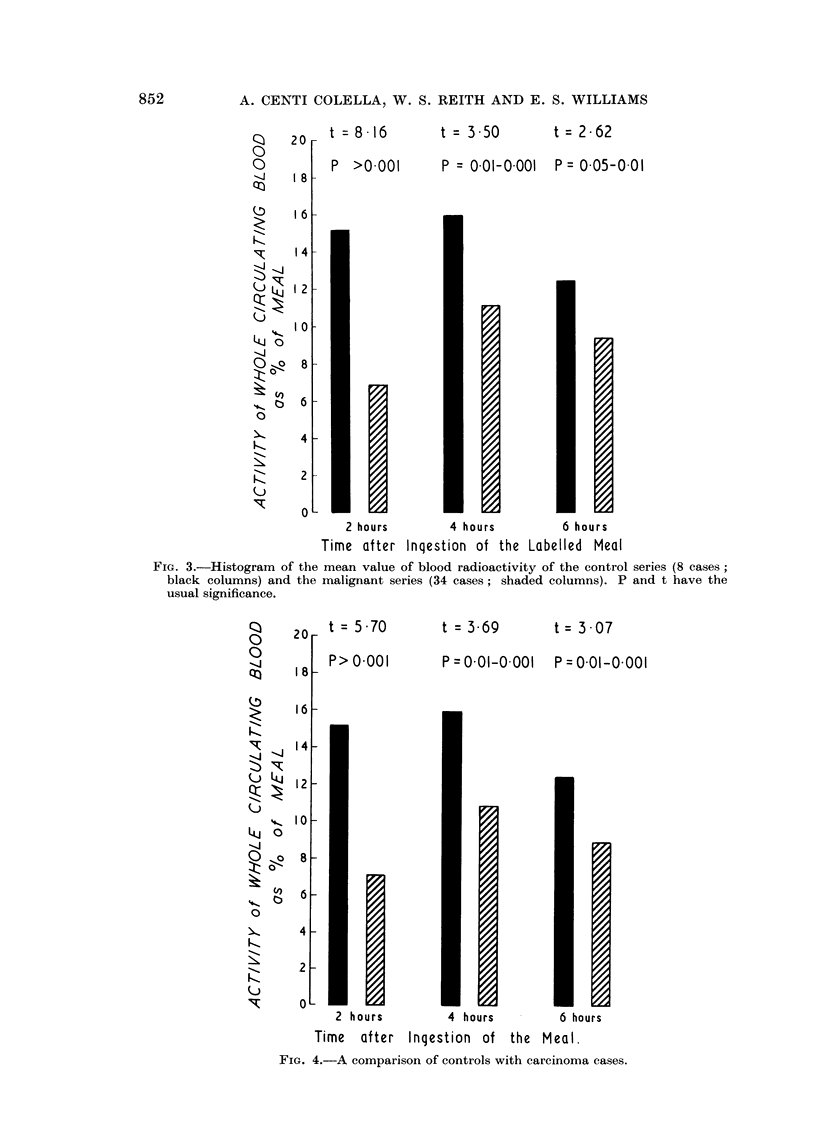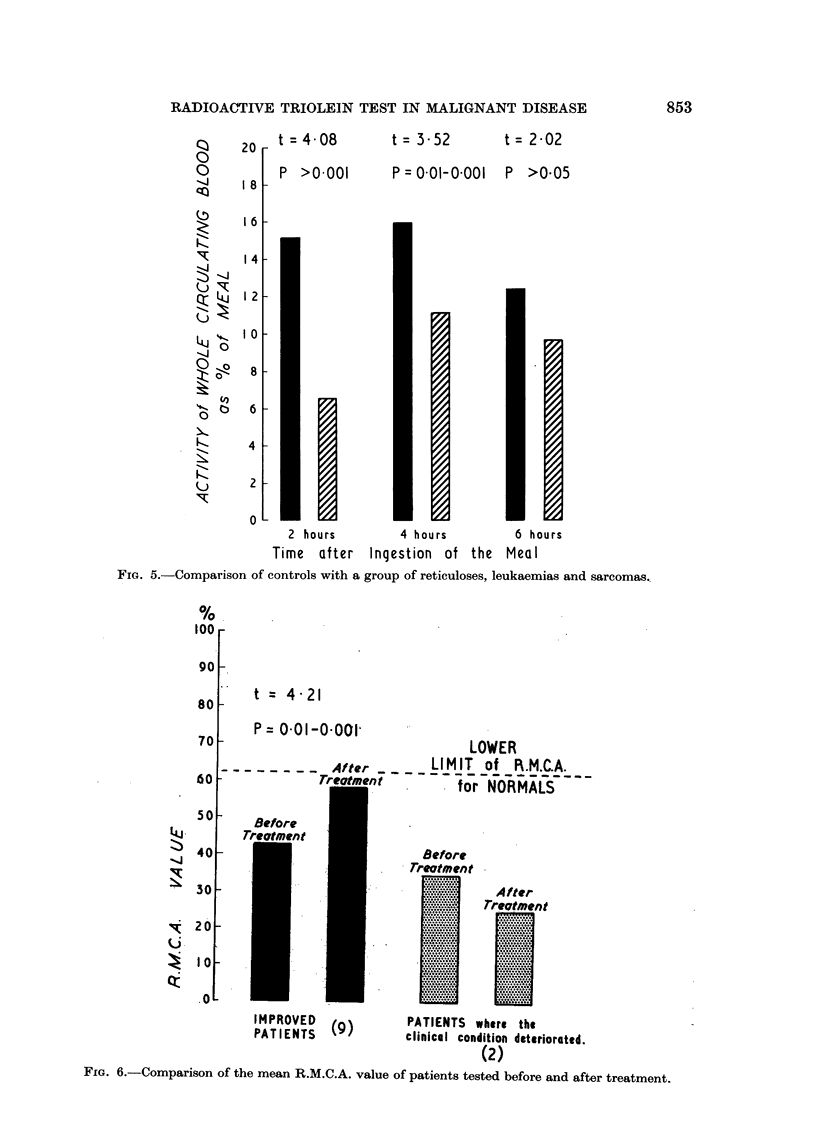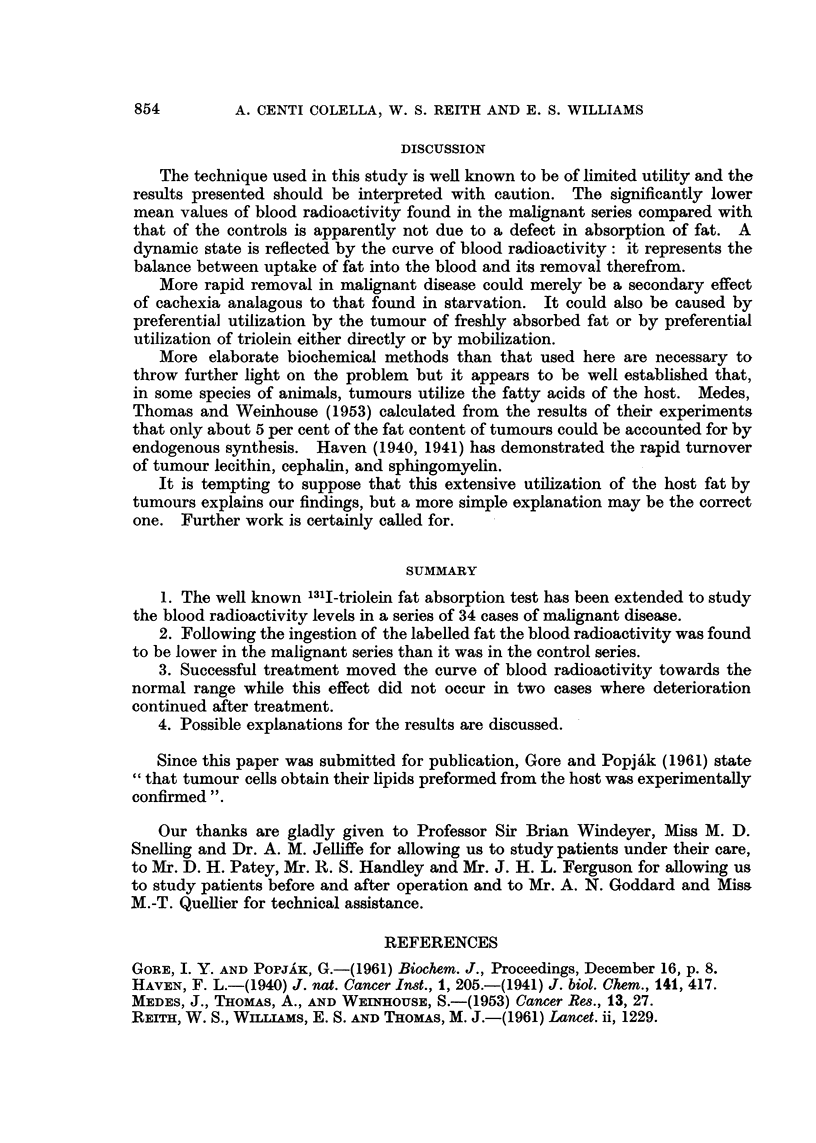# The Radioactive Triolein Test in Malignant Disease

**DOI:** 10.1038/bjc.1961.98

**Published:** 1961-12

**Authors:** A. Centi Colella, W. S. Reith, E. S. Williams


### THE RADIOACTIVE TRIOLEIN TEST IN MALIGNANT DISEASE

A. CENTI COLELLA,* W. S. REITH AND E. S. WILLIAMS

*From the Institute of Nuclear Medicine, Middlesex Hospital Medical School, London, W.1*

Received for publication August 1, 1961

REITH, WILLIAMS AND THOMAS (1961) have reported results using a standardised technique for the $^{131}$I-triolein fat absorption test. In studying how the presence of diseases other than those involving fat malabsorption affected the results, it was observed that the level of blood radioactivity was below the normal range when malignant disease was present.

This observation has now been studied in 34 cases, brief details of these being given in Table I. Patients with tumours of the gastro-intestinal tract were excluded from the series. The age range was 13–89 years and there were 22 males and 12 females.

### METHODS

Details of the preparation and standardisation of the labelled fatty meal are given by Reith *et al.* (1961). Thyroid uptake of $^{131}$I was blocked by the administration of Lugol's solution on each of the two days preceding the test and on the day of the test the patient fasted overnight and ingested the labelled meal at about 9 a.m., and thereafter remained fasting until about noon, when food and fluid was allowed to be taken normally. Venous blood samples were taken at 2, 3, 4 and 6 hours after ingestion of the meal and faeces were collected 24 hourly until the faecal radioactivity had fallen approximately to background level.

The results of the tests were referred to those from a group of 8 normal subjects. The mean value of blood radioactivity at 2, 4 and 6 hours after ingestion of the meal was compared with the corresponding mean value in the whole malignant series, in the connective tissue tumour series, and in the carcinoma series, by applying Student's t test. A similar statistical comparison was made in a group of patients on whom the study was made both before treatment, and after tumour regression and clinical improvement had followed treatment.

### RESULTS

The radioactivity contained in the total circulating blood in a group of normal subjects is shown in Fig. 1.

The broken line in Fig. 1 represents the mean circulating radioactivity and the shaded area one standard deviation on either side of the mean. Intercomparison between patients is difficult because of the irregular shape of the curves, so in each case the area enclosed by the curve, the abscissa, and the 6-hour ordinate was calculated and expressed as fraction of the similar area bounded by the curve of the mean normal plus one standard deviation (upper limit of the shaded area). This fraction was called the " relative manipulation curve area " (R.M.C.A.).

* Fellow of the International Atomic Energy Agency. Present address : Isttiuto di Radiologia Medica dell' Universita di Roma, Italy.

TABLE I.—*Experimental Data of the Malignant Series and of the Control Series*

| Patients | Sex | Age | Activity of whole circulating blood as % of meal: 2 hours | 4 hours | 6 hours | R.M.C.A. |
|---|---|---|---|---|---|---|
| *Group of normals* | | | | | | |
| B | M. | | 18·2 | 14·2 | 11·8 | 83·4 |
| C | ,, | | 7·8 | 20·0 | 7·4 | 69·0 |
| D | F. | | 24·7 | 15·6 | 15·4 | 104·2 |
| E | M. | | 14·0 | 17·6 | 15·5 | 84·8 |
| I | ,, | | 18·7 | 14·7 | 13·9 | 87·2 |
| N | F. | | 13·4 | 13·5 | 10·6 | 70·0 |
| BF | ,, | | 13·9 | 16·0 | 12·7 | 81·5 |
| 2BF | ,, | | 10·7 | 16·0 | 12·1 | 74·6 |
| *Malignant neoplasms* | | | | | | |
| BY | F | 44 | 1·3 | 6·4 | 7·3 | 24·5 |
| CH | ,, | 49 | 9·1 | 9·5 | 8·4 | 48·6 |
| CI | ,, | 69 | 8·6 | 14·0 | 9·3 | 65·8 |
| CO | M | 19 | 6·4 | 7·4 | 7·7 | 37·2 |
| AC | ,, | 13 | 0·7 | 13·9 | 9·0 | 41·6 |
| AE | ,, | 31 | 7·8 | 9·1 | 7·3 | 45·4 |
| AN | ,, | 23 | 7·0 | 18·3 | 9·5 | 65·3 |
| AI | ,, | 59 | 4·7 | 9·2 | 8·6 | 39·7 |
| BA | F. | 63 | 4·2 | 9·2 | 9·4 | 39·2 |
| AF | M. | 38 | 7·7 | 12·5 | 6·2 | 50·6 |
| CT | ,, | 74 | 9·0 | 15·2 | 11·7 | 65·8 |
| CU | ,, | 62 | 5·1 | 18·0 | 14·3 | 62·0 |
| CX | ,, | 54 | 6·4 | 7·8 | 13·7 | 46·0 |
| CY | ,, | 74 | 11·7 | 11·9 | 18·1 | 67·4 |
| BW | F | 78 | 9·5 | 10·5 | 10·1 | 54·5 |
| BX | ,, | 66 | 4·6 | 6·3 | 5·8 | 29·8 |
| CA | ,, | 74 | 8·1 | 8·6 | 8·2 | 45·3 |
| BZ | ,, | 86 | 5·5 | 21·5 | 16·5 | 77·2 |
| CC | M. | 70 | 6·4 | 13·8 | 8·4 | 53·0 |
| CD | F. | 67 | 8·1 | 17·2 | 12·1 | 67·4 |
| CF | ,, | 57 | 7·0 | 13·1 | 10·0 | 53·7 |
| CG | ,, | 81 | 7·7 | 9·7 | 8·2 | 46·6 |
| CK | M. | 53 | 7·1 | 8·9 | 8·6 | 43·9 |
| CL | F. | 67 | 12·0 | 13·4 | 10·5 | 66·0 |
| CM | M. | 69 | 5·4 | 8·1 | 10·2 | 40·3 |
| AX | ,, | 69 | 8·6 | 11·7 | 10·0 | 54·5 |
| F | ,, | 71 | 10·0 | 10·3 | 7·7 | 42·4 |
| AZ | ,, | 79 | 8·0 | 11·7 | 8·6 | 52·5 |
| AV | ,, | 26 | 6·6 | 10·9 | 5·7 | 43·8 |
| AY | ,, | 64 | 3·9 | 7·3 | 3·8 | 28·1 |
| CP | ,, | 79 | 4·6 | 6·9 | 5·9 | 31·4 |
| CQ | ,, | 64 | 2·7 | 9·3 | 11·4 | 38·5 |
| CR | ,, | 46 | 7·3 | 8·4 | 8·2 | 43·6 |
| CZ | ,, | 63 | 10·3 | 9·1 | 9·2 | 52·0 |

A histogram of the R.M.C.A. values of the patients studied in the present series is shown in Fig. 2. In all except three patients the faecal radioactivity is in the normal range suggesting that depression of the blood radioactivity curve is not due to defective absorption of fat.

Although eight patients in the malignant series had an R.M.C.A. value in the normal range statistical analysis of the difference between the mean of the malignant group and of the normal group gave the significance detailed in Fig. 3. The experimental data are recorded in Table I.

The malignant series was subdivided into (*a*) carcinomas and (*b*) other tumours and reticuloses and an analysis similar to that above was carried out, the result being shown in Fig. 4 and 5.

In 11 cases the test was carried out before and after the treatment. In 9 of these regression of the tumour and clinical improvement had occurred at the

FIG. 1.—Blood radioactivity, faecal radioactivity, and results of chemical fat balance studies in a group of normal subjects.

time of the second test while in 2 cases the clinical condition had deteriorated. The data referring to these tests are shown in Table II and the comparison of the results is shown in Fig. 6.

We have not found any statistically evident influence on the R.M.C.A. values of the age of the patients (over 64 years and under), the rate of tumour growth, differentiation, or the tumour volume roughly calculated from clinical notes or X-ray evaluations.

FIG. 2.—A histogram of the R.M.C.A. values and the faecal radioactivity obtained in a series of normal patients and in a series with malignant disease.

TABLE II.—*Experimental Data of Patients Tested Before and After Treatment*

| Patients | Treatment | R.M.C.A. Before | R.M.C.A. After |
|---|---|---|---|
| BY | DXR-therapy | 24·5 | 27·6 |
| CF | Mastectomy | 53·7 | 72·4 |
| AC | DXR-therapy | 41·6 | 57·7 |
| CM | $^{60}$Co-therapy | 40·3 | 46·8 |
| CO | $^{60}$Co-therapy | 37·2 | 51·6 |
| CR | DXR-therapy | 43·6 | 50·7 |
| AE | $^{60}$Co-therapy | 45·4 | 78·8 |
| AF | $^{60}$Co-therapy | 50·6 | 81·2 |
| CH | Chemotherapy | 48·6 | 46·8 |
| BX | DXR-therapy | 29·8 | 23·5 |
| BA | Chemotherapy | 39·2 | 26·4 |

FIG. 3.—Histogram of the mean value of blood radioactivity of the control series (8 cases; black columns) and the malignant series (34 cases; shaded columns). P and t have the usual significance.

FIG. 4.—A comparison of controls with carcinoma cases.

FIG. 5.—Comparison of controls with a group of reticuloses, leukaemias and sarcomas.

FIG. 6.—Comparison of the mean R.M.C.A. value of patients tested before and after treatment.

### DISCUSSION

The technique used in this study is well known to be of limited utility and the results presented should be interpreted with caution. The significantly lower mean values of blood radioactivity found in the malignant series compared with that of the controls is apparently not due to a defect in absorption of fat. A dynamic state is reflected by the curve of blood radioactivity : it represents the balance between uptake of fat into the blood and its removal therefrom.

More rapid removal in malignant disease could merely be a secondary effect of cachexia analagous to that found in starvation. It could also be caused by preferential utilization by the tumour of freshly absorbed fat or by preferential utilization of triolein either directly or by mobilization.

More elaborate biochemical methods than that used here are necessary to throw further light on the problem but it appears to be well established that, in some species of animals, tumours utilize the fatty acids of the host. Medes, Thomas and Weinhouse (1953) calculated from the results of their experiments that only about 5 per cent of the fat content of tumours could be accounted for by endogenous synthesis. Haven (1940, 1941) has demonstrated the rapid turnover of tumour lecithin, cephalin, and sphingomyelin.

It is tempting to suppose that this extensive utilization of the host fat by tumours explains our findings, but a more simple explanation may be the correct one. Further work is certainly called for.

### SUMMARY

1. The well known $^{131}$I-triolein fat absorption test has been extended to study the blood radioactivity levels in a series of 34 cases of malignant disease.

2. Following the ingestion of the labelled fat the blood radioactivity was found to be lower in the malignant series than it was in the control series.

3. Successful treatment moved the curve of blood radioactivity towards the normal range while this effect did not occur in two cases where deterioration continued after treatment.

4. Possible explanations for the results are discussed.

Since this paper was submitted for publication, Gore and Popják (1961) state " that tumour cells obtain their lipids preformed from the host was experimentally confirmed ".

Our thanks are gladly given to Professor Sir Brian Windeyer, Miss M. D. Snelling and Dr. A. M. Jelliffe for allowing us to study patients under their care, to Mr. D. H. Patey, Mr. R. S. Handley and Mr. J. H. L. Ferguson for allowing us to study patients before and after operation and to Mr. A. N. Goddard and Miss M.-T. Quellier for technical assistance.

### REFERENCES

GORE, I. Y. AND POPJÁK, G.—(1961) *Biochem. J.*, Proceedings, December 16, p. 8.
HAVEN, F. L.—(1940) *J. nat. Cancer Inst.*, **1**, 205.—(1941) *J. biol. Chem.*, **141**, 417.
MEDES, J., THOMAS, A., AND WEINHOUSE, S.—(1953) *Cancer Res.*, **13**, 27.
REITH, W. S., WILLIAMS, E. S. AND THOMAS, M. J.—(1961) *Lancet*. ii, 1229.